# Part of speech tagging of grammatical features related to L2 Chinese development: A case analysis of *Stanza* in the L2 writing context

**DOI:** 10.3389/fpsyg.2023.1139703

**Published:** 2023-02-15

**Authors:** Ge Lan, Xiaofei Pan, Yachao Sun, Yuan Lu

**Affiliations:** ^1^Department of English, City University of Hong Kong, Hong Kong, Hong Kong SAR, China; ^2^Language and Culture Center, Duke Kunshan University, Suzhou, China; ^3^Department of Asian and Slavic Languages and Literatures, The University of Iowa, Iowa City, IA, United States

**Keywords:** part of speech tagging, SLA, corpus linguistics, language development, grammatical features, Chinese as a second language

## Abstract

Grammatical complexity has received extensive attention in second language acquisition. Although computational tools have been developed to analyze grammatical complexity, most relevant studies investigated this construct in the context of English as a second language. In response to an increasing number of L2 Chinese learners, it is important to extend the investigation of grammatical complexity in L2 Chinese. To promote relevant research, we evaluated the new computational tool, *Stanza*, on its accuracy of part-of-speech tagging for L2 Chinese writing. We particularly focused on eight grammatical features closely related to L2 Chinese development. Then, we reported the precisions, recalls, and F-scores for the individual grammatical features and offered a qualitative analysis of systematic tagging errors. In terms of the precision, three features have high rates, over 90% (i.e., *ba* and *bei* markers, classifiers, -*de* as noun modifier marker). For recall, four features have high rates, over 90% (i.e., aspect markers, *ba* and *bei* markers, classifiers, -*de* as noun modifier marker). Overall, based on the F-scores, Stanza has a good tagging performance on *ba* and *bei* markers, classifiers, and -*de* as a noun modifier marker. This evaluation provides research implications for scholars who plan to use this computational tool to study L2 Chinese development in second language acquisition or applied linguistics in general.

## 1. Introduction

The recent two decades have witnessed an expanding application of computational techniques in empirical studies on second language acquisition (SLA). Grammatical complexity has been considered one of the major indices of language development. Multiple computational tools have been designed and applied to support grammatical analyses, for instance, the Second Language Syntactic Complexity Analyzer (Lu, 2010) and the Tool for the Automatic Analysis of Syntactic Sophistication and Complexity (Kyle, 2016). The applications of these tools effectively improve research efficiency in investigating grammatical complexity and language development. These tools have been employed in corpus studies, where a large amount of language data often needs to be processed. Studies have shown the usefulness of these tools in SLA research (e.g., Lu, 2011; Kyle and Crossley, 2018); however, most of these studies have been conducted in the English as a second language (ESL) context. Since a growing number of learners have begun to study Chinese as a second language (L2), there is an urgent need to explore how computational tools can help examine L2 Chinese development from the perspective of grammatical complexity. Multiple computational tools have been used to analyze the Chinese language, and we chose the most recent computational package to explore (i.e., Stanza), which was just developed and released by Qi et al. (2020). Since in SLA, grammatical complexity has been found closely associated with language development; we then focused on testing the Stanza’s part-of-speech (POS) tagging accuracy on grammatical features closely associated with L2 Chinese development. Following the conventions of tagging evaluation (Pustejovsky and Stubbs, 2013), this study reports precision, recall, and F-score for each selected grammatical feature. Specific suggestions are then provided to illustrate how to use Stanza to investigate these grammatical features. This study can potentially leverage the application of computational techniques in L2 Chinese research.

## 2. Literature review

### 2.1. Grammatical analysis and language development

The research of grammatical complexity has a long history in applied linguistics. The notion of *complexity* has received much research attention since the 1990s in multiple fields, including but not limited to natural sciences, social sciences, and humanities (Bulté and Housen, 2012). Grammatical complexity has been considered one of the core constructs due to its crucial roles, for example, benchmarking language development (Ortega, 2012). In applied linguistics, some terms such as linguistic complexity, syntactic complexity, and grammatical complexity, to a certain extent, are interrelated. Among them, linguistic complexity is an umbrella term that includes multifaceted language use sources, such as morphological features, lexical features, syntactic structures, and grammatical features (Bulté and Housen, 2012). Under this umbrella term, syntactic complexity and grammatical complexity have been both frequently investigated in applied linguistics, but scholars have used the concepts in different senses. For example, syntactic complexity has been used to describe the complexity based on the construction of syntactic structures (e.g., the number of clauses per T-unit). In contrast, grammatical complexity has been adopted to describe complexity depending on the use of individual grammatical features (e.g., adjectives and adverbial clauses). We include both grammatical complexity and syntactic complexity in this section to ensure the comprehensiveness of reviewing the existing studies about language development. As a result, we use the term, grammatical complexity, to refer to the studies of both syntactic structures and grammatical features.

Grammatical complexity has been found effective in reflecting L2 writing development. Such studies are based on two major ways of operationalizing “development,” which are (1) a cross-sectional design to compare grammatical complexity features (and/or measures) in written productions of L2 learners across different academic levels and (2) a longitudinal design to compare the features (and/or measures) in written productions of L2 learners across different periods of time.

In the cross-sectional and longitudinal research, most grammatical complexity studies have been conducted to analyze ESL development, and other languages (including Chinese) have received less attention. In terms of cross-sectional studies, Lu (2011) applied 14 grammatical measures (e.g., complex nominals per T-unit) to investigate L2 English development across four different school levels in universities. Lu (2011) found good “candidates” (i.e., the measures) that can distinguish school levels, including but not limited to coordinate phrases per T-unit, mean length of clauses, and complex nominals per T-unit. Then, Parkinson and Musgrave (2014) compared the use of noun modifiers (e.g., attributive adjectives, relative clauses) in academic essays between ESL learners and L2 master’s students. They found ESL students used more attributive adjectives, a basic noun modifier, whereas the master’s students produced more advanced modifiers (e.g., relative clauses and prepositional phrases as post-modifiers). More recently, Ansarifar et al. (2018) compared the use of phrasal complexity features (e.g., premodifying noun, prepositional phrases) in English research abstracts written by Iranian MA students, Iranian PhD students, and expert writers. Their findings suggest that in general, the PhD students produced phrasal features in a similar way as the expert writers; However, the MA students produced fewer phrasal features in their writing. For the longitudinal studies, Bulté and Housen (2014) compared multiple measures (e.g., mean length of noun phrase) in ESL essays written by students at the beginning and end of a 4-month semester. Some measures are found effective to capture the short-term language development in academic writing, such as the mean length of clauses and the mean length of noun phrases. Gray et al. (2019) explored the ESL development of Chinese learners based on clausal and phrasal features in TOEFL iBT tasks, with both written and spoken tasks. Biber et al. (2020) recently conducted a study to track students’ language development over a 2-year period based on specific lexico-grammatical features (e.g., relative clauses). Both studies demonstrate that phrasal features (e.g., premodifying nouns, prepositional phrases as post-modifiers) capture language development in academic writing.

As mentioned above, most empirical studies have been conducted on English, and other languages have received much less attention (Lan et al., 2019). The exploration of grammatical features in relation to the development of L2 writing in non-English languages are still limited, e.g., Spoelman and Verspoor (2010) on L2 Finnish, Vyatkina (2013) and Vyatkina et al. (2015) on L2 German, Hu (2021) on L2 Chinese. Thus, there is a need to promote the relevant studies about other languages than English in applied linguistics.

### 2.2. Computational tools and grammatical analysis

One of the most important reasons for the booming of grammatical analysis in L2 writing research is the successful development and application of computational tools. For example, a computational tool that has been frequently used is the Second Language Syntactic Complexity Analyzers (L2SCA). This tool was developed by Lu (2010) to automate the calculation of 14 large-grained syntactic measures, which have been found effective in capturing ESL development in previous milestone studies (i.e., Wolfe-Quintero et al., 1998; Ortega, 2003). These measures cover five important grammatical types: length of production (e.g., mean length of clauses), subordination (e.g., clauses per T-unit), coordination (e.g., coordinate phrases per clause), sentential complexity (i.e., clauses per sentence), and particular structures (e.g., complex nominals per T-unit). The L2SCA has facilitated empirical research on grammatical complexity in ESL writing, for example, Lu (2011), Yang et al. (2015), Yoon (2017), Yoon and Polio (2017), Eckstein and Ferris (2018), Casal and Lee (2019), and Lan et al. (2022a). Another popular computational tool that is used in register studies is Biber Tagger, developed by Biber (1988) to tag the form and function of a wide range of lexico-grammatical features (e.g., attributive adjectives, -ing clauses as post modifiers). The Biber Tagger has been widely used in empirical studies on grammatical complexity in L2 writing, such as Lan et al. (2022b). Different from Lu’s (2010) L2SCA, the Biber Tagger helps investigate individual lexico-grammatical features. Another tool for the Automatic Analysis of Syntactic Sophistication and Complexity (TAASSC) is a relatively new one developed by Kyle (2016). This tool includes numerous fine-grained measures for clausal complexity (e.g., adjective complements per clause), noun phrase complexity (dependents per indirect object), syntactic sophistication [e.g., average faith score verb (cue)–construction (outcome)–news], and the 14 syntactic measures in Lu (2010). For its young age, TAASSC has not been applied as much as other tools in empirical studies, and one of a few examples is Kyle and Crossley (2018). Nevertheless, this tool has the potential to be applied more frequently in the future due to the comprehensiveness of the fine-grained measures.

Scholars in computational linguistics have also built other tools for grammatical analysis. One of the most prestigious tools is the Stanford CoreNLP, which applies multiple linguistic annotations for text analysis and linguistic annotation, including but not limited to word tokenization, sentence tokenization, part-of-speech (POS) tagging, named entities recognition, and syntactic parsing. The tool is considered stable for natural language processing. The Stanford CoreNLP was designed for English, and then the development team has expanded its functions to other languages, such as French, Spanish, Arabic, Japanese, and Chinese. In addition, the Natural Language Toolkit (NLTK) is a set of libraries in Python for processing human languages (Bird et al., 2009). In addition to the diverse functions, such as tokenization, POS tagging, and syntactic parsing, NLTK offers numerous corpora and useful lexical resources (e.g., WordNet). Another user-friendly tool is the Tree Tagger, which can annotate texts with lemma and POS information (Schmid, 1994). The Tree Tagger has been used to annotate a set of human languages, including but not limited to German, English, Italian, Russian, and Chinese. Next, the TagAnt is a valuable POS tagging tool with a user interface. It is one of the computational tools developed by Laurence Anthony in the recent decade (2011–present). This tool, a POS tagger, can process several languages (e.g., English, Spanish, and French). As this tool has a very user-friendly interface, scholars without much computational background can easily use it to process their data. Last, one of the most recent computational tools, Stanza, is developed by a group of computational linguists from Stanford University (Qi et al., 2020). Stanza as a Python toolkit allows users to annotate 60+ languages, and it is well-tested with high-accuracy neural network components. We need to acknowledge that the list of computational tools can never be exhausted, and we only included some commonly used tools in applied and computational linguistics.

These tools support interdisciplinary research among computational linguistics, corpus linguistics, and applied linguistics. This is especially the case for research on grammatical complexity in SLA. Finally, we need to point out that the tools have primarily been trained based on a large amount of general language data rather than data in specific genres. For instance, Stanza was trained based on a mixed use of Common Crawl (i.e., general website archives), Wikipedia Dumps (i.e., Wikipedia online data in general), and Google One-billion Word (Qi et al., 2020). The training based on the general language data allows these tools to perform well in diverse situations. Having said this, scholars in applied linguistics tend to be more interested in analyzing language use in specific genres (e.g., argumentative papers, research reports, exam tasks) because the tools often have different performances in different genres. For example, perhaps due to the consideration of genre influence, Picoral et al. (2021) recently focused on evaluating three tools in English argumentative papers, an important genre in composition courses at university.

### 2.3. Studies on L2 Chinese grammatical development in writing and the limited application of computational tools

Chinese SLA has a keen interest in grammar development, which helps build a solid body of L2 Chinese grammar research (Lu and Ke, 2018). Lu and Ke’s (2018) synthesized primary studies during the past few decades involving a wide array of grammatical features from function words (e.g., adverbs and aspect markers), phrases (e.g., nominal structures and verbal complements), clauses (e.g., relative clauses), to discourse-related speech units (e.g., cohesive devices and information structures). These features have long attracted research and pedagogical foci in L2 Chinese because they present various and idiosyncratic learning difficulties concerning form, function, and form–function mapping. Although the investigation of these features contributes to our knowledge of acquisition orders, production rate and accuracy, processing difficulties, crosslinguistic influences, and sociolinguistic factors underlying learners’ development of those discrete grammatical features, relevant studies seldom include multiple related features from the perspective of grammatical complexity in relation to L2 Chinese development in writing.

On the other hand, research on grammatical complexity has burgeoned in the area of L2 Chinese writing, initially driven by the need for indices addressing typological characteristics of Chinese (e.g., topic-comment units and zero-anaphora clauses) to measure writing quality and development (Jin, 2007; Jiang, 2013; Yu, 2021) and then furthered by investigations of more linguistically-motivated finer-grained indices (e.g., Wu, 2016, 2018; Pan, 2018, 2023; Hu, 2021; Wu and Lu, 2021; Hu et al., 2022; Lu and Wu, 2022).

Attempting to include a large set of grammatical features in a single study, Pan (2018) derived a battery of 21 frequency-based indices from 210 essays of two topics/genres written by 105 tertiary L2 Chinese learners across four course levels and seven writing quality levels. The indices represented grammatical complexity at various levels, i.e., global (T-unit length), clausal (paratactic and hypotactic clauses), phrasal (complex noun phrases) complexity, rank shifted constituents (e.g., clauses functioning as object) as well as specific grammatical features unique in Chinese (e.g., *ba*- and *bei*-constructions). The results showed that while most of the indices showed little differences between adjacent course levels, complex noun phrases using relative clauses and total complex verbs (including 10 varieties of verbal modifiers and complements) had more discriminative power for essay quality and course levels. Then, Pan (2023) focused on three types of complex nominal structures, i.e., epithet/classifier modifiers, relative clauses, and *act clauses* that function like nominal heads (Halliday and Matthiessen, 2014), and described their developmental features in 40 essays on the same topics as in Pan (2018) written by 20 L2 Chinese learners from three course levels at a Sino-U.S. joint-venture university. The results indicated that complex nominal structures increased with the course level, and relative clauses played a critical role in developing and enriching the variety of complex nominal structures. The significant role of complex noun phrases in L2 Chinese writing has been explored by Wu and Lu (2021) and Lu and Wu (2022) to examine the relationships between three noun-phrase-based indices and three topic-comment (TC)-based indices regarding the quality of narrative and argumentative essays written by L2 Chinese learners at a Chinese university. The results showed minimal correlations between the TC indices and the essay scores, in contrast to the medium effect sizes of the ratio of complex noun phrases and the total length of all complex premodifiers, which explained the variance in narrative and argumentative essay scores, respectively. The findings also indicated that fine-grained indices were more useful than large-grained indices to (1) capture the internal properties of single clauses, (2) be less affected by genres, and (3) discriminate writing quality for learners with a similar proficiency level.

Until very recently, the improvement in grammatical feature coverage and annotation efficiency with automatic annotation tools for Chinese texts was demonstrated by Hu and Xiao (2019), who used LTP-Cloud (Che et al., 2010) to build two large-scale corpora and extracted nine types of phraseological units in Chinese (i.e., collocations such as verb-object and adjective-noun combinations). Based on Hu and Xiao’s (2019) work, Hu (2021), and Hu et al. (2022) compared the discriminative power of a set of phraseological indices vs. indices based on T-units and TC-units, respectively, for a large corpus of learners’ writing for HSK, the official standardized L2 Chinese proficiency test in China, and a smaller institutional dataset of learners’ writing. As for Hu et al. (2022), while the TC indices still needed to be derived manually, the automatic annotation tool much more efficiently derived 21 indices to measure the diversity, sophistication, and complexity of eight phraseological features in the dataset. Group-wise comparisons and correlational tests showed that multiple phraseological indices (e.g., root ratios of predicate-comment combinations) exhibited stronger correlations with writing quality and/or larger effects for discriminating learners’ proficiency levels. Specifically, the language-independent features (e.g., verb-object combinations) were found to discriminate better between the beginning and intermediate levels, while the Chinese-unique features (e.g., predicate-comment combinations) discriminated better between the intermediate and advanced levels. Moreover, predicate-centered modifications (e.g., adverbials) were suggested to contribute more to L2 Chinese writing than nominal modifications (e.g., classifiers).

All these reviewed L2 Chinese studies echo SLA researchers’ argument for encompassing multiple grammatical features in single studies (Lu and Ke, 2018; Zhang and Tao, 2018) and investigating grammatical complexity from multiple dimensions, i.e., global, clausal, and phrasal levels as well as features that are sensitive to interlanguage development and conceptual demands of tasks (Norris and Ortega, 2009; Robinson et al., 2009; Biber et al., 2011; Lan et al., 2022a), for which POS serves as the basis. Several computational tools automatically processing English texts have contributed significantly to the rapid increase and advancement of studies on grammatical complexity in relation to ESL learners’ writing development/quality; however, counterpart studies in L2 Chinese have primarily relied on labor-intensive manual annotation in SLA. Thus, an urgent methodological consideration remains to find accessible and reliable computational tools to empower POS annotation for L2 Chinese learners’ writing samples, especially those unique features in the Chinese language.

**FIGURE 1:**
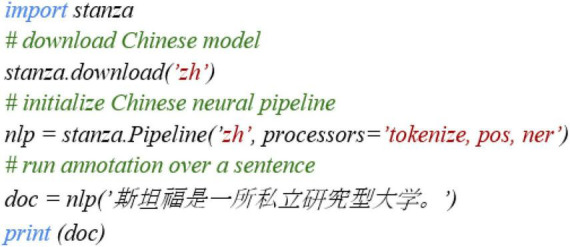
Sample codes in Qi et al. (2020), CC-BY 4.0.

Among the computational tools that can process the Chinese language, this study evaluates Stanza for basic POS annotation, particularly those grammatical features that have received the most research interest in L2 grammar development in SLA (Lu and Ke, 2018). The reasons to choose Stanza for analysis include (1) *Recency*: It is the most recent tool that provides POS tagging for the Chinese language, which was just released in 2020 (Qi et al., 2020); (2) *Accessibility*: Stanza is an open-source Python toolkit that can be publicly accessible; (3) *Ease of use*: The use of this tool is fairly straightforward and easy, with only five-line Python codes, as Qi et al. (2020) demonstrated (see Figure 1); (4) *Reported accuracy*: Qi et al. (2020) mentioned Stanza’s performance in the Chinese language (i.e., The F-score of POS tagging is 88.93%, with the treebank-specific tagset). However, this performance is based on L1 Chinese texts, so we do not know much about its tagging on L2 Chinese texts. Also, the performance is holistic, and it is unclear whether the tool has high accuracy in tagging individual grammatical features closely related to L2 Chinese development in SLA. We consider that SLA scholars might be more interested in the accuracy of tagging these features instead of a holistic tagging accuracy in general; (5) *Genre effects*: Similar to most tools, Stanza has been trained based on online databases (e.g., Google One-billion Words) reflecting general language use, but SLA scholars might be interested in specific genres in their studies. Thus, our evaluation is based on the two common but important written genres in tertiary L2 Chinese courses.

However, we need to acknowledge that (1) Stanza’s tagging can be improved with algorithm training, but scholars in SLA may not have the advanced computational techniques to do it, and most scholars perhaps only apply the tools designed as they are; (2) Based on the reported accuracy performance in Qi et al. (2020), other existing tools may have better performance to annotate the Chinese language such as Stanford CoreNLP and Language Technology Platform. The evaluation of Stanza does not aim to argue for substituting other tools with Stanza but to explore if Stanza, as a very recent, open-source, easily applicable computational package, can be used as an additional tool to supplement grammatical complexity research on L2 Chinese development. Therefore, such an evaluation would provide preliminary but useful implications for SLA scholars and applied linguists in general.

We address three research questions based on L2 Chinese writing:

1. What are the precision rates of Stanza for selected grammatical features?
2. What are the recall rates of Stanza for selected grammatical features?
3. What are the F-scores of Stanza for the selected grammatical features?

## 3. Methods

### 3.1. Corpus building

A corpus of L2 Chinese writing was built based on 40 academic papers from 20 L2 Chinese learners at a Sino-US joint-venture university. The participating students were from three course levels that target the intermediate, the upper intermediate, and the advanced proficiency levels, respectively, according to the program’s course descriptions. Two writing tasks were sent to the students to generate their written productions, with one prompt for expository writing and the other for picture descriptions. The two written genres were selected because they are not only common genres in academic writing but also the major genres that students need to handle in Chinese courses.

Table 1 demonstrates the basic information of the corpus, which includes two subcorpora: (1) one for expository essays (20 files), with 6,623 tokens and around 331 Chinese characters per file; (2) another for picture descriptions (20 files), with 4,573 tokens and about 228.65 Chinese characters per file. In general, the two subcorpora were comparable. The original data in this corpus was then cleaned, removing irrelevant information (e.g., student names, page numbers). Then, the original files (in Word format) were converted into plain text files with AntFile converter (Anthony, 2022). Finally, a Python program was run to adjust the format further, which included deleting additional spaces, removing empty lines, and adjusting the line breaks. By the end of this step, the corpus was ready for further analysis.

**TABLE 1:** Description of the corpus.

Corpus	File number	Total tokens	Average length
Expository essay	20	6,623	331.15
Picture description	20	4,573	228.65

Tokens refer to Chinese characters for this table.

### 3.2. Target grammatical features

As reviewed in the previous sections, grammatical complexity is multidimensional, and POS is the basis for all levels of complexity. Based on these considerations as well as Stanza’s tagging capacity, eight features were selected in this study: common verbs, common nouns, classifiers, *-de* as noun modifier markers, adverbs, aspect markers, and *ba* and *bei* markers. This list is by no means comprehensive due to the limited scope of this study, but the target features (1) have attracted much attention in the existing studies on L2 Chinese grammar development as synthesized in Lu and Ke (2018); (2) include grammatical features that are unique in Chinese. Table 2 presents eight grammatical features to be evaluated, which can be directly extracted based on the POS tags. With a combination use of these POS tags, we can extract a wider range of grammatical features, for example, using an adjective-noun sequence to extract attributive adjectives. However, the combination use of the POS tags is not the focus of our study, and we only narrowed it down to the manageable set of these eight features. Thus, the accuracy of their corresponding POS tags generated by Stanza can be directly useful in assessing the targeted grammatical features of L2 Chinese texts.

**TABLE 2:** Description of target grammatical features.

Target features in this study	Stanza POS tags	Relevance to L2 Chinese grammar development as in Y. Lu and Ke (2018)	Relevance to L2 writing development	Examples
Common verbs	VV	Verbal complements	Complex verb phrases	*bù kěnéng **chī** wán* “impossible to finish eating”
Common nouns	NN	Nominal structures	Complex noun phrases	*yì tái **diànshì** bèi **rén** ná zǒu le* “a TV set was taken by someone”
Classifiers	NNB	Classifiers	Complex noun phrases; unique grammatical features in Chinese	*yí **gè** nán háizi* “a young guy”
*-de* as noun modifier marker	DEC	Relative clauses; associatives	Complex noun phrases	*xǐhuān **de** shū; māma zuò **de** fàn* “book that (someone) likes”; “meals that Mom makes”
Adverbs	RB	Adverbs	Adverbs/adverbials (that modify verb phrases and clauses)	*wǒ **yě hěn** xǐhuān chūqù chī fàn* “I also much like going out to eat”
Aspect markers	AS	Aspect markers	Complex verb phrases	*yí gè nán kàn **le** tā* “a man has seen him”
*ba* marker *bei* marker	BB BB	The *ba*-constructions The *bei*-constructions	Unique grammatical features in Chinese Unique grammatical features in Chinese	*tā **bǎ** yí gè fāngxíng de dōngxi ná chūlái* “he takes out a square-shaped thing” *yì tái diànshì **bèi** rén ná zǒu le* “a TV set was taken away by someone”

Target features in underlined and bold Pinyin.

### 3.3. The application of Stanza

Stanza was a recently developed computational tool in 2020. Qi et al. (2020) introduce Stanza as an open-source Python toolkit that provides multiple types of linguistic annotations, including tokenization and POS tagging. As Stanza is a Python toolkit, a Python program was used to conduct the POS tagging on our corpus. Our program was built based on the sample code provided in Qi et al. (2020) (see Figure 1 below):

The input of the Python program is the 40 plain text files in the corpus. The program output was the tagged files in CONLL format (see Appendix for a tagged sample).

### 3.4. Evaluation of POS tagging

The evaluation of POS tagging refers to calculating the accuracy of POS tags assigned by Stanza in this study. There are two types of errors that a POS tagger can make, namely “places where it put the wrong tag on an item, and places where it failed to put the right tag” (Pustejovsky and Stubbs, 2013, p.173). For example, when the target feature for checking was adverb in L2 Chinese writing, the two types of errors could be: (1) Stanza assigned an adverb tag to a word that was not an adverb; (2) Stanza failed to assign an adverb tag to a word, which was, in fact, an adverb. The first type of error could be shown by calculating the precision (i.e., the number of correct tags of an item out of the total number of tags of this item), and the second type of error could be indicated by calculating the recall (i.e., the number of assigned tags of an item out of the actual number of this item). To evaluate the overall performance, F-scores were calculated based on “the harmonic mean of the precision and recall of an algorithm’s performance over a tag” (Pustejovsky and Stubbs, 2013, p.175).

To operationalize the calculation of the precision and recall, computational linguists often apply the confusion matrix (Bird et al., 2009; Pustejovsky and Stubbs, 2013). Table 3 shows the important terms associated with the calculation of precision, recall, and F-score. Gold tags refer to the tags manually assigned by human coders, which are considered 100% correct. In this study, two human coders (two PhDs in SLA with a research emphasis on L2 Chinese) manually added gold tags to the corpus. Machine tags refer to the tags automatically assigned by Stanza. According to the relationship between gold tags and machine tags, there are four different situations, namely true positive (tp), false positive (fp), false negative (fn), and true negative (tn). To clarify the four conditions, we presented the four corresponding examples based on the adverb tagging in our corpus:

**TABLE 3:** The confusion matrix.

	Machine tags	
Gold tags	True positive (tp)	False negative (fn)
	False positive (fp)	True negative (tn)

1. True positive (tp): A true adverb is assigned by an adverb tag.
2. False positive (fp): A true adverb is not assigned by an adverb tag.
3. False negative (fn): A non-adverb is assigned by an adverb tag.
4. False positive (fp): A non-adverb is not assigned by an adverb tag.

Three formulae were provided to calculate the precision, recall, and F-score: (1) Precision = tp/(tp + fp); (2) Recall = tp/(tp + fn); (3) F-score = (2*Precision*Recall)/(Precision + Recall). Thus, the precisions, recalls, and F-scores were calculated for all the targeted grammatical features by another two human coders with research experience in corpus linguistics.

## 4. Results

The evaluation of Stanza’s POS tagging on precision, recall, and F-scores were separately reported in the expository essays, the picture descriptions, and all files in the corpus. The precision rates, recall rates, and F-scores were classified into two groups: (1) the rates greater than 90% were considered high rates, and (2) the rates lower than 90% were considered low rates. We need to acknowledge that there is no specific cut-off value to differentiate between high and low rates of POS tagging in SLA or applied linguistics in general. Some existing studies used 90% as a benchmark for POS tagging (e.g., Lan and Sun, 2019; Lan et al., 2019). In these studies, no manual adjustment was conducted to fix POS tags if precision, recall, and F-score were higher than 90%. Following this convention, we used 90% as the cut-off value in this study.

### 4.1. The precision rates

The precision rates of the target features are reported in Table 4. In general, the precision rates for the two different tasks are close to each other in the expository essays, picture descriptions, and all files in the corpus, except for the aspect markers. Three features have high precision rates (>90%). The *ba* and *bei* markers have the highest precision rates among all the target features, which are 100% for the expository essays, 96% for the picture descriptions, and 98% for all files in the corpus. Then, *-de* as noun modifier markers has the second highest precision rates: 97.4% for the expository essays, 94.1% for the picture descriptions, and 95.8% for all files in the corpus. Similarly, classifiers have high precision rates, although the rates are not as ideal as *ba* and *bei* markers and -*de* as noun modifier markers. The classifiers have 90% for the expository essays, 94.7% for the picture descriptions, and 95.8% for all files in the corpus.

**TABLE 4:** Precision rates of the target features.

Target features	Essay	Picture	All files
Adverbs*	0.621	0.595	0.608
Common verbs*	0.839	0.859	0.849
Common nouns*	0.895	0.871	0.883
Aspect markers*	0.474	0.960	0.717
*Ba* and *bei* markers	1.000	0.960	0.980
Classifiers	0.900	0.947	0.923
-*de* as noun modifier marker	0.974	0.941	0.958

*Marks the target features with one or more precision rates lower than 90%.

In contrast, four target features have low precision rates (<90%). Adverbs are the target features with the lowest precision rates, 62.1% for the expository essays, 59.5% for the picture descriptions, and 60.8% for all files in the corpus. Then, common verbs and common nouns also have low precision rates, but these rates can still be considered close to the cut-off value (90%). Common nouns and common verbs, respectively, have 89.5 and 83.9% for the expository essays, 87.1 and 85.9% for the picture descriptions, and 88.3 and 84.9% for all files in the corpus. The outlier in the evaluation of the POS tagging precision is aspect markers because this feature has 47.4% for the expository essays but 96% for the picture descriptions. Overall, the precision rate is 71.7% for all the files.

### 4.2. The recall rates

The recall rates of the target features are presented in Table 5. Similar to precision, the recall rates for the two writing tasks are also close in the expository essays, the picture descriptions, and all files in the corpus. However, one exception is adverbs. Four target features have high recall rates (>90%). Two target features have perfect recall rates (100%) for the expository essays, the picture descriptions, and all files in the corpus, which are *ba* and *bei* markers and aspect markers. This is much higher than any of the remaining target features. The other two features have high recall rates as well, although the rates are not perfect: (1) *-de* as a noun modifier marker has recall rates over 99%, namely 99.6% for the expository essays, 99.1% for the picture descriptions, and 99.3% for all files in the corpus; (2) classifiers have the recall rates over 95%, which are 96.9% for the expository essays, 95.9% for the picture descriptions, and 96.4% for all files in the corpus.

**TABLE 5:** Recall rates of the target features.

Target features	Essay	Picture	All files
Adverbs*	0.927	0.693	0.810
Common verbs*	0.829	0.894	0.862
Common nouns*	0.801	0.862	0.832
Aspect markers	1.000	1.000	1.000
*Ba* and *bei* markers	1.000	1.000	1.000
Classifiers	0.969	0.959	0.964
-*de* as noun modifier marker	0.996	0.991	0.993

*Marks the target features with one or more recall rates lower than 90%.

In terms of recall rates, three target features have low rates (<90%). For recall, common verbs are the least problematic among the three features. The recall rates for the expository essays, the picture descriptions, and all files in the corpus are 82.9, 89.4, and 86.2%, respectively. In addition, common nouns also have recall rates greater than 80%, which are 80.1% for the expository essays, 86.2% for the picture descriptions, and 83.2% for all files in the corpus. Finally, there is also an outlier, adverbs, whose recall rates are very different in the two tasks, with a high rate (92.7%) for the expository essays and a low rate (69.3%) for the picture descriptions.

### 4.3. The F-scores

The F-scores of the target features can be found in Table 6. F-score is an overall evaluation based on precision and recall rates. Three target features have high F-scores, which are *ba* and *bei* markers, classifiers, and -*de* as a noun modifier marker. This is within expectation because the three features have high precision and recall rates, leading to high F-scores. *Ba* and *bei* markers have the highest F-scores, 100% for the expository essays, 98% for the picture descriptions, and 99% for all files in the corpus. Then, classifiers have the F-scores greater than 93% (i.e., 93.3, 95.3, and 94.3%), and -*de* as a noun modifier marker have the F-scores greater than 96% (i.e., 98.5, 96.5, and 97.5%), for the expository essays, the picture descriptions, and all files in the corpus.

**TABLE 6:** F-scores of the target features.

Target features	Essay	Picture	All files
Adverbs*	0.744	0.640	0.695
Common verbs*	0.834	0.876	0.855
Common nouns*	0.845	0.866	0.856
Aspect markers*	0.643	0.980	0.835
*Ba* and *bei* markers	1.000	0.980	0.990
Classifiers	0.933	0.953	0.943
-*de* as noun modifier marker	0.985	0.965	0.975

*Marks the target features with one or more F-scores lower than 90%. The F-score for all the target features in all the files of the corpus is 87.8%.

In terms of the low F-scores, common verbs and common nouns have similar F-scores, relatively higher than adverbs and aspect markers. The F-scores of common verbs are 83.4% for the expository essays, 87.6% for the picture descriptions, and 85.5% for all files in the corpus. Then, the F-scores of common nouns are not much different, 84.5% for the expository essays, 86.6% for the picture descriptions, and 85.6% for all files in the corpus. However, the remaining two features have some issues regarding F-scores. Adverbs have the lowest F-scores compared to others, which are only 74.4% in the expository essays, 64.0% in the picture descriptions, and 69.5% in all files. The issue of aspect markers is not caused by low F-scores but by the inconsistency of the F-scores between the expository essays (64.3%) and the picture descriptions (98%).

## 5. Discussion

### 5.1. Similarities and differences from the developers’ report

Qi et al. (2020) developed Stanza and provided an overall evaluation of Stanza’s POS tagging. It is important to clarify a major difference between our evaluation and Qi et al.’s (2020) evaluation of the POS tagging function. Qi et al. (2020) trained, tested, and evaluated the Stanza based on L1 Chinese texts, whereas our evaluation is based on texts produced by L2 Chinese learners. In addition, we only focus on evaluating target grammatical features closely related to L2 Chinese development, as reviewed by Lu and Ke (2018). While acknowledging the importance of evaluating Stanza with L1 Chinese texts, which provides Stanza users an overall understanding of how this computational tool performs, we consider that the evaluation of L2 Chinese data can greatly benefit scholars in SLA. Despite this difference, our evaluation and Qi et al.’s (2020) evaluation can be considered similar. Qi et al. (2020) reported an F-score of 88.93% to indicate the overall performance of POS tagging (for treebank specific tags, i.e., XPOS tags). We also calculated the F-score for all the target grammatical features: 87.84%. This value is slightly lower but very close to Qi et al.’s (2020) F-score. A tentative conclusion is that Stanza has a comparable POS tagging performance for L1 and L2 Chinese data, although more evaluations should be conducted to validate this point. Also, we need to mention that Qi et al. (2020) did not report precision and recall rates, so we cannot discuss these two values in this study.

### 5.2. Reasons behind the high and low rates

Our results suggest that the grammatical features can be classified into a high-rate group (e.g., *ba* and *bei* markers) and a low-rate group (e.g., adverbs). The evaluative results are mostly consistent across the expository essays, the picture descriptions, and all files in the corpus. Regarding the reasons behind this, our analysis below is primarily from the perspective of the Chinese language instead of technical aspects (e.g., training algorithms). Due to the limited space of this paper, the discussion only focuses on systematic tagging errors of Stanza (denoted by “*”), whereas random tagging errors caused by learners’ mistakes in writing (e.g., typos, lexical and grammatical errors, mixing foreign words) are not included in the discussion. The English translation for the examples only indicates the meaning of the Chinese words instead of their grammatical counterparts.

#### 5.2.1. BB: *ba* and *bei* markers

Stanza combines *ba* and *bei* makers into the BB tag. The *ba*-construction is a unique grammatical feature in Chinese that indicates the affectedness and disposal of an object caused by an action. *Ba*-construction changes the canonical SVO word order of Chinese in that it requires the direct object/noun phrase (marked by *ba*) to precede the verb phrase, as shown in Box 1. This unique construction may contribute to the perfect precision and recall for *ba* in both expository essays and picture descriptions.

**BOX 1. Example of Ba.**

**Table:** 

	t*ā*	bǎ	yí g$\overset{`}{e}$ fangxíng de d$\bar{o}ngx\bar{ı}$
	Subject or agent	*ba*	Noun phrase (direct object or patient)
	He	*ba*	A square-shaped thing
	n$\overset{´}{a}$	ch$\bar{u}l\overset{´}{a}$i
	Verb	Other element
	Take	Come out
	He takes out a square-shaped thing

The *bei*-construction with the direct object in the sentence-initial position expresses passive meanings in Chinese, following the structure: the direct object/noun phrase 1 plus *bei* followed by an optional agent/noun phrase 2 and a verb phrase (Li and Thompson, 1989) as displayed in Box 2.

**BOX 2. Example of Bei.**

**Table:** 

	yì t$\overset{´}{a}$i di$\overset{`}{a}$nshì	b$\overset{`}{e}$i	(r$\overset{´}{e}$n)
	Noun phrase 1	bei	Noun phrase 2 (optional agent)
	(direct object or patient)
	A TV set	bei	Person
	n$\overset{´}{a}$	zǒu
	Verb	Other element
	Take	Walk
	A TV set is taken away (by someone).

The only erroneous BB tag was found in one picture description where a verb inherently entailing adversary meaning *bèipò* (“forced”), which was wrongly parsed into *bei*, the passive marker and a component of non-verb (*bèi* BB/*pòzuò VV/*huàishì VV “forced to do bad things”). Interestingly, Stanza also wrongly tokenizes the direct object of the verb phrase to be another verb. Although it is an error for this instance, the structural formula applies to a *bei*-construction with an unexpressed agent, as shown in Box 2. The multi-functioning feature of *bei* to serve stand-alone as a passive marker and to compose a verb with adversary connotation (Lü, 1999) may contribute to the tagging error.

#### 5.2.2. NNB: Classifiers

In Chinese, a classifier denotes the property of the associated noun (Gobbo, 2014) and must occur with a number (e.g., *yī* “one”), a demonstrative (e.g., *zhè* “this”), or certain quantifiers (e.g., *jǐ* “how many”) before a head noun (Li and Thompson, 1989). Measure words for length (e.g., *mǐ* “meter”), weight (e.g., *bàng* “pound”), and for occurrences of an event (e.g., *cì* “time”) are also counted as classifiers (Li and Thompson, 1989). In addition, Chinese grammar allows nouns such as containers (e.g., *píng* “bottle”), body parts (e.g., *shēn* “body”), and time (e.g., *fēnzhōng* “minute”) to serve as “borrowed” or quasi-measure words (Liu et al., 2001).

The fixed construction of noun phrases with classifiers may lead to a high rate of precision and recall of the NNB tag. The tagging errors are mainly caused by (1) overgeneralization of non-NNB words that happen to be between a cardinal number and a noun (e.g., *yìdiǎn cài* “a little dish” is mistagged as *yī* CD “cardinal number, one”/*dian NNB/*cài* NN “dish”), (2) failure to specify borrowed measure words especially when the head nouns are omitted (e.g., *mǎi yì pán*, should be interpreted as to buy a plate NNB of food instead of to buy a plate NN), (3) confusion of *gè* as a component of a common noun (e.g., *gèrén* individual person) with the most common classifier *gè* (e.g., *yí gè rén* “one *ge* person”), and (4) mis-combination of a demonstrative and a classifier into one single word (e.g., *měi gè DT “every”/*xīngqī* NN “week”).

#### 5.2.3. DEC: *-de* as noun modifier marker

The particle *-de* links associative phrases (i.e., noun phases) and modifying phrases (e.g., a relative clause or an attributive adjective) before a head noun (Li and Thompson, 1989). Although the Penn Chinese Treebank 3.0 (Xia, 2000) distinguishes *-de* added after associative phrases and modifying phrases with two tags, DEG and DEC, Stanza applies DEC as a generic tag to include both types of prenominal modifiers. Despite the high precision and recall rates, Stanza seems to systematically confuse (1) *-de* (的) DEC for *-de* (地) DEV (adverbial particle) when *-de* (的) happens to be followed by a verb phrase (e.g., *wéiyī* “only”/*de DEV/*chī* VV “eat”/*zhūròu* “pork”/*de* DEC/*rén* “person” “the only person that eats pork”), and (2) *-de* (的) DEC with *de* (的) UH (the sentence-final particle, with no hyphen) (e.g., *tā* “he”/*gāogāo* “tall-tall”/*de DEC “he is tall”).

Regarding the second confusion, the construction of reduplicated adjective plus the sentence-final particle *de* functions as the predicate in a sentence to enhance the vividness of what is being described, which is a unique grammatical feature in Chinese grammar (Liu et al., 2001). A further look into the only one correct tagging among a total of four such constructions shows that the period mark used immediately after *de*, indicating the end of the sentence overtly, may help Stanza successfully recognize *de* as UH. Moreover, given the increasing research interest in the role of noun phrases in writing (e.g., Lan et al., 2022a; Lu and Wu, 2022; Pan, 2023) and L2 Chinese learners’ different developmental paths of *-de* in associative and modifying phrases (Zhang, 2002), we suggest that Stanza distinguishes DEG and DEC as described in the Penn Chinese Treebank 3.0 to improve the identification of fine-grained linguistic features.

In contrast, the following sub-sections discuss features in the low-rate group (i.e., adverbs, common verbs, and common nouns) and aspect markers with mixed results.

#### 5.2.4. RB: Adverbs

An adverb occurs before a verb or an adjective, modifying and defining the action, behavior, quality, and status involved in a sentence or the whole sentence (Liu et al., 2001). Stanza’s mistagging of adverbs is systematic across five categories.

First, conjunctions such as *dànshì* “but,” *kěshì* “but,” suǒyǐ “therefore,” *érqiě* “moreover,” *lìngwài* “in addition,” *ér* “and/but,” *búguò* “however,” *ránhòu* “and then,” and *cóng’ér* “so that,” share the tag RB with adverbs. However, although adverbs and conjunctions overlap in the grammatical function of clause linking, they belong to different word classes and indicate different levels of grammatical complexity. Thus, we suggest that Stanza uses distinct tags for adverbs, coordinating, and subordinating conjunctions as described in the Penn Chinese Treebank 3.0.

Second, as a precision issue, a few demonstratives, prepositions, and particles are mistagged as adverbs. Demonstratives *zhème* “like this,” *nàme* “like that,” and *zěnme* “how” followed by a verb or an adjective are all tagged as adverbs (e.g., *zhème RB/*guì* VA “expensive like this”). Preposition *lián* in the *lián*……*yě/dōu*…… construction that singles out one part of the sentence with the meaning “even” (Li and Thompson, 1989, p. 338) is all tagged as an adverb (e.g., *lián RB/*yí gè rén* “one person”/*yě* RB/*méiyǒu* VV “there is not even a single person”). Particles *lái*, *ér*, and *suǒ* that appear before a verb are identified as MSP (other particles) in the Penn Chinese Treebank 3.0 but are all tagged as adverbs by Stanza (e.g., *huā hěn cháng shíjiān* “spend long time”/*lái RB/*zuòfān* “make meals”). In contrast, as a recall issue, adverb *yǒudiǎnr* “a little bit,” including its variations *yǒuyìdiǎn* and *yǒudiǎn*, are either mistagged as VV or split into the verb *you* “have” and *(yì)diǎn* inconsistently mistagged as RB, JJ, or CD plus NNB.

Third, adjective reduplication that serves as adverbials for verb phrases was split into RB plus another tag (e.g., *qiāo RB “sneaky”/*qiāo VV “sneaky”/*de* DEV/*tōu* VV “steal” …… “sneakily steal ……”). However, constructions like *qiāoqiāo de* “sneakily” are “adverbial forms of the adjectival verb and express features of manner, such as ‘quickly’ or ‘carefully”’ (Halliday and McDonald, 2004, p. 317) instead of adverbs. Given the needs, many Chinese words can serve various parts of speech without any morphological inflection (c.f., in English, *sneaky* is an adjective and *sneakily* is an adverb). For example, *jí* “haste,” *kuài* “fast,” and *gāoxìng* “happy” can serve as attributives, adverbials, or complements in sentences (Liu et al., 2001). Therefore, these words should remain as adjectives when being tagged.

Moreover, Stanza seems to fail to detect grammatically multi-functioning words such as *zài* that serve as an adverb expressing actions in progress (e.g., *wǒ* “I”/*zài* RB/*chīfàn* “eat meal” “I am eating a meal”), a preposition indicating location or temporality (e.g., *wǒ* “I”/*zài* “at”/*fànguǎn* “restaurant”/*chī fàn* “eat meal” “I eat at a restaurant”), or an existential verb (e.g., *wǒ* “I”/*zài* “being at somewhere”/*fànguǎn* “restaurant” “I am located at a restaurant”) in different contexts. Adverb *zài* is normally followed by a verb phrase, which is structurally different from the preposition *zài* and the verb *zài* that are often followed by a noun phrase. Stanza successfully tags six instances of adverb *zài* whereas missing the other three. In two of the three wrong instances, Stanza also mistags the verb phrase following *zài* as NN (e.g., *fāxiàn* “discover”/*tā* “he”/*zài VV/*dǎo NN “to play (tricks)”/*de* DEC/*guǐ* “trick” “discover the trick he played”).

Lastly, when a monosyllabic adverb, such as *bù, zuì, jiù, gēng, hěn, tài, zhī, dōu*, and *zhēn*, is used before a monosyllabic adjective, copula, common verb, or another adverb, Stanza tends to count the construction as a single word (e.g., *bùhǎo* JJ “not good” instead of *bù* RB “negator”/*hǎo* JJ “good”). In contrast, the same adverb followed by a disyllabic word is identified as RB plus another tag (e.g., *bù* RB/*xǐhuān* VV “not to like”). Word parsing for written Chinese is challenging and sometimes debatable because the natural boundaries in written Chinese are between *zì* Chinese characters instead of *cí* words. Chinese characters are monosyllabic and mostly have individual meanings themselves, which is prominent in classical Chinese and influential till today, co-existing with the disyllabic tendency of modern Chinese (Li and Thompson, 1989; Lü, 1999). This influence may explain Stanza’s tendency to combine a monosyllabic adverb and a monosyllabic adjective or verb into a disyllabic word, especially those highly frequent collocates such as *hěn hǎo* “very good,” *zuì hǎo* “most good, i.e., best,” *bù hǎo* “not good.”

#### 5.2.5. VV: Common verbs

Verbs describe events, actions, states, processes (change of states), and experiences (Li and Thompson, 1989). Despite the clear semantic features of verbs in Chinese, the structural boundaries between verb compounds and verb phrases as well as word-class transfers and grammatical multi-functioning without morphological marking often make controversial parsing and tagging results.

It is debatable whether some frequent disyllabic verb-object (e.g., *liáotiān* “chat”) and subject-predicate constructions (e.g., *tóuténg* “headache”), some of which are also known as *separable verbs* (离合词), are verb compounds or phrases. According to Li and Thompson (1989), a verb compound has one or more of these features: (1) one or both of the constituents being bound morphemes, (2) idiomaticity of the meaning of the entire unit, or (3) limited separability of the constituents. However, it is arguable whether those constructions tagged as VV by Stanza, such as *chīfàn* “eat meal,” *shàngkè* “attend class,” *huíjiā* “return home,” *kāichē* “drive vehicle,” *zǒulù* “walk,” and *fùqián* “pay money,” are verb compounds or phrases.

Another controversy comes in the tagging for resultative verb compounds (RVC). Li and Thompson (1989) describe RVC as “a two-element compound” in which “the second element signals some result of the action or process conveyed by the first” (p. 54–55) (e.g., *dǎpò* “break something and make it broken”), including their potential form (e.g., *dǎbúpò* “break something but not able to make it broken”). Stanza selectively tags RVC as VV when the second element is monosyllabic such as *pò* “broken,” *suì* “shattered,” *dào* “arrive,” and *wán* “complete,” whereas splits the construction if the second element is polysyllabic (e.g., *ná* VV “take”/*chūlái* VV “come out” “take out”) including their potential form (e.g., *kàn* VV “look”/*bù* AD “negator”/*qīngchǔ* VA “clear” “not able to see clearly”).

As for word-class transfers, while some of the tagging errors are obvious given the syntactic structure, e.g., *gōngzuò* “work” that serves a noun in *méiyǒu gōngzuò* “have no work” is mistagged as VV, it is not so obvious to judge whether *ānjìng* “quiet” in *suǒyǒu dōu ānjìng le*” all have become quiet” is a verb or an adjective, or whether *pínghéng* “balance” in *yígè xīngqī liǎng cì chūqù chī fàn shì pínghéng de* “eating out twice a week is balanced” a verb, an adjective, or a noun.

Stanza’s tagging errors are also found in grammatically multi-functioning words such as *zài* and *gěí*. Besides mistagging adverb *zài* as VV as previously discussed, Stanza inconsistently tags preposition (IN) *zài*, which is the majority of over 250 total instances of *zài*, as IN correctly and VV incorrectly. Similarly, a total of ten instances of *gěí* are all tagged as VV, among which three are true verbs (e.g., *gěí* VV “give”/*fànguǎn* “restaurant”/*shēngyi* “business”) whereas seven should be prepositions (e.g., *gěí VV/*jingchá* “police”/*da* “call”/*diànhuà* “telephone” “call the police”).

Moreover, we suggest that Stanza consistently includes verb reduplication [e.g., *shì(yi)shì* VV “try-try, i.e., have a try”] in the VV category, and tags verb negation as a negative adverb, *bù* or *méi*, plus a verb (e.g., *méi* RB/*kàn* VV “have not seen”) instead of a single verb (e.g., *méikàn* VV “have not seen”).

#### 5.2.6. Common nouns

Like the word-class-transfer-related tagging error of VV, a few nouns should not be tagged as NN when they have transferred to serve as adjectives and verbs given the syntactic structures that they are in (e.g., *bù* RB “negator”/*dàodé NN “moral”/*de* DEC/*háizi* NN “kid” “immoral kid”; *jianchá NN “check”/*shìfou* “whether or not”/*you* “have”/*rén* “people” “to check whether anyone is there”).

Word boundaries concerning prefixes and suffixes (nominal morphemes) and localizers (a sub-class of noun) also impact tagging for common nouns. First, although Chinese word classes are not marked morphologically, several nouns distinguish themselves by prefixes or suffixes that precede or follow a root morpheme (Liu et al., 2001). In Chinese, prefixes are extremely rare, while suffixes are slightly more numerous (Li and Thompson, 1989). Our data include only a few common nominal suffixes such as -*yuán* “member of a profession or group,” -*zú* “person belonging to a clan,” -*xìng* “possessing some property,” -*men* “plural form for people,” and -*sù* “possessing some element.” Except for those true suffixes, most units tagged as PFA (prefix) and SFN (suffix) are constituents of nominal compounds or phrases (e.g., *túshū* NN/*guan SFN “library,” *xiao PFA/*nánhái* NN “little boy”). Moreover, disyllabic nouns like *fànguǎn* “restaurant” and *rénmen* “people” are more likely to be tagged as NN whereas polysyllabic nouns like *túshūguan* “library” and *péngyǒumen* “friends” as NN plus SFN. However, since prefixes and suffixes are bound morphemes, it seems hierarchically problematic to single out prefixes/suffixes as if they are paralleled to root morphemes that are tagged as NN.

Second, localizers (LC), such as *shàng* “top,” *yǐshàng* “up from,” and *shàngbiān* “top side” indicate directional and spatial relations (Liu et al., 2001). While most nominal modifiers precede a head noun, localizers are the only post-nominal modifiers, which turn a common noun to a place that can be used after prepositions like *zài* “at” and *dào* “until” (e.g., *zài* IN/*jiā* NN/*lǐ* LC “at home”). Besides, all localizers, except those monosyllabic ones, can be used individually as head nouns by themselves (e.g., *shàngbiān* LC “top side”/*yǒu* “have”/*rén* “person” “there is someone at the top”) (Li and Thompson, 1989). Although the Penn Chinese Treebank 3.0 includes LC, Stanza does not use LC but counts localizers in the category of IN and tags those constructions composed of a non-monosyllabic noun followed by a localizer as NN plus IN (e.g., *zài* IN/*zhè zhāng zhàopiàn* NN “this piece of photo”/*shàng IN “top” “on this piece of photo”) and other constructions composed of a monosyllabic NN and a monosyllabic LC as NN (e.g., *zài* IN/*jiā lǐ NN “home inside”/*chī fàn* “eat meals” “eat at home”).

#### 5.2.7. AS: Aspect markers

Aspect indicates different ways of viewing a situation and reflects the concept of temporality (Robinson et al., 2009), especially in the absence of tense in Chinese grammar. Stanza tags three aspect markers: *-le*, the perfective marker that indicates viewing of an event in its entirety, *-zhe*, the imperfective marker that indicates the ongoing duration of action, and *-guo*, the experiential marker that indicates a situation having been experienced (Li and Thompson, 1989). Stanza’s overall precision and recall rates for aspect markers are very high, except for the extraordinarily low precision for the expository essays. We speculate that the overall low frequency of aspect markers in the expository essays amplifies the impact of Stanza’s confusion of *guò* (过 VV “to spend”) with -*guo* (过 AS) and *le* (了 UH indicating the change of status) with *-le* (了 AS) on the precision rate.

Among the four instances of 过 in the expository essays, Stanza successfully identifies one AS (*xǐhuān* VV “like”/-*guo* AS/*le* UH) and one VV (*zěnme* “how”/*guò* VV/*de* UH) but mistags the other two VV as AS (e.g., *guò AS/*shēngrì* “birthday” “to celebrate the birthday”). As for the picture descriptions, *-guo* AS occurs five times, among which three are correctly tagged (e.g., *qù* VV “go”/-*guo* AS/*túshūguǎn* “library” “have been to the library”) whereas the other two are mistakenly treated as part of the preceding verb (e.g., *qùguò VV/*-le* AS/5 *gōngli* “5 kilometers” “been away for 5 kilometers”). As for 了, we again observed the influence of punctuation marks because only the correct instance includes the period mark after *le* (i.e., *è sǐ* “starve to death”/*le*_°_ UH “become starving to death”). In contrast, other punctuation marks or words are used after *le* in the remaining three instances where *le* is mistagged as AS (e.g., ……/*tài hao* “too good”/*le AS/*yīnwéi* “because”…… “something is too good because ….”).

There are some other tagging errors caused by random incorrect parsing and idiosyncratic tagging inconsistency of the same words, which are not discussed here due to the focus and space of the paper. In summary, Stanza’s errors can be ascribed to parsing errors and tagging errors. Specifically, the parsing errors are related to (1) the absence of natural boundaries between Chinese words in writing, (2) the dual identity of some constructions that can be used as word compounds and phrases, and (3) the diachronic change from monosyllabic classical Chinese to disyllabic modern Chinese. On the other hand, the tagging errors are related to (1) polysemous words that can have multiple different functions/meanings and (2) the lack of one-to-one correspondence between Chinese words and word classes. These are the challenges for developing computational tools for processing the Chinese language.

## 6. Conclusion, implications, and limitations

This study reports the evaluation of Stanza’s POS tagging on L2 Chinese data, a corpus of L2 Chinese writing, with expository essays and picture descriptions. Our evaluation is based on the grammatical features that are closely related to L2 Chinese development, a vital research construct in SLA. The evaluation shows that the *ba*- and *bei*- markers, classifiers, and -*de* as noun modifier markers have high precision rates, recall rates, and F-scores. In contrast, adverbs, common verbs, and common nouns have low precision rates, recall rates, and F-scores. The evaluative results are consistent in the two types of L2 Chinese writing, but the only exception is aspect markers. The reasons for the evaluative results are interpreted after a qualitative corpus analysis. Specific examples from the corpus are provided.

This study also provides research implications for SLA scholars who are interested in using Stanza for their research. Without a computational tool that automatically and reliably tags POS for Chinese texts, the labor-intensive manual annotation may hinder further exploration of L2 Chinese grammatical complexity in writing, especially the derivation of fine-grained linguistic features in large-size corpora. Stanza serves as a promising tool for SLA-Chinese researchers to alleviate the manual labor of annotation to semi-automatic tagging and expand their research scope. However, manual adjustment is inevitable depending on the target features. Minor manual adjustment on the POS tags is needed for the group of features with high rates. We suggest using Stanza to tag L2 Chinese texts for grammatical analysis. Scholars can search the corresponding POS tags (i.e., NNB for classifiers, DEC for *de* as a noun modifier marker, and BB for *ba* and *bei* markers) in the tagged corpora to automatically retrieve these grammatical features. This is particularly convenient for scholars who want to explore L2 Chinese development based on these grammatical features. Since Stanza does not distinguish *ba* and *bei* markers despite their distinct language forms, researchers need to differentiate the *ba* and *bei* constructions manually or with an additional Python script. In contrast, more meticulous manual adjustment would be needed for the tags for adverbs, common verbs, and common nouns. These three features are in the group of low rates, and the overall tagging performance, indicated by their F-scores (69.5, 85.5, and 85.6%), might be lower than expected (90%). SLA-Chinese scholars can focus on the problematic cases (e.g., tagging errors caused by grammatical multifunctioning and word-class transfers) that we identified in the discussion, which is likely to help fix the tags efficiently. Finally, it is surprising that aspect markers have inconsistent precision rates in two types of writing. This might be due to the different topics for the two writing tasks. Particular attention should be paid to the writing topics in SLA research when Stanza is used to tag L2 Chinese written texts.

This study is not without limitations. First, in terms of the term, “grammatical complexity,” we need to acknowledge that “grammatical complexity” and “syntactic complexity” are conceptually not the same. We use “grammatical complexity” to make our discussion more comprehensive and to align with the POS tagging on lexico-grammatical features. Next, as an exploratory study that still involves intensive manual annotation, our corpus only consists of 40 L2 Chinese writings, and the corpus size is much smaller than large-scale corpora used for tool evaluation in computational linguistics. We acknowledge that increasing the corpus size will provide a more generalized evaluation. Also, the eight target features in this study do not cover comprehensively all meaningful grammatical features in relation to L2 Chinese development due to the lack of either use in the corpus or clear definition of the tags. Other features such as prepositions, connectives and conjunctions, and attributive and predicative adjectives are worth examination in future studies. All in all, POS resides at the base level of the hierarchy of grammatical complexity. The results of POS tagging should be used as the smallest building blocks that researchers combine and connect to construct the level(s) of grammatical complexity for purposeful investigation.

As for the tool, Stanza POS tags are labeled as treebank-specific tags in Qi et al. (2020), but it is not entirely consistent with the Chinese tagset of the Penn Treebank project. As we confirmed with one of the Stanza developers, the treebank-specific tagset turns out to be a combination of partial Chinese tagset and partial English tagset of the Penn Treebank project. In this case, some grammatical features cannot be clearly defined. Common verbs and common nouns in Stanza’s treebank-specific tagset do not perfectly align with the definitions in the Chinese tagset of the Penn Treebank project. After a pilot tag checking with the two scholars in SLA and Chinese language education, we defined common verbs as all non-copular verbs and common nouns as all non-proper nouns. Moreover, our qualitative analysis does not cover all tagging errors but only those systemic errors with observable patterns and controversial cases are inevitable. We welcome other scholars to validate our way of defining the target features in the future. Next, it is important to point out that precision, recall, and F-scores can be influenced by the accuracy of word tokenization. The overall accuracy of word tokenization was 91.2% for all the files in the corpus. We need to acknowledge that since the tokenization accuracy is over 90%, we did not fix the tokenization errors in the tagged corpus. Finally, we need to acknowledge that POS tagging is only one of the functions that Stanza provides, and other functions can also support grammatical analysis, for instance, syntactic parsing. We call for further studies to evaluate other functions to explore better pathways for doing grammatical analysis for L2 Chinese. This will be helpful for SLA scholars who are interested in analyzing grammar usage in L2 Chinese writing.

## Data availability statement

The raw data supporting the conclusions of this article will be made available by the authors, without undue reservation.

## Author contributions

GL contributed to the corpus tagging, program building, and the calculation of precisions, recalls, and F-scores of the lexico-grammatical features. XP contributed to the corpus building, the gold labels of the lexico-grammatical features, and the analysis of the (dis)advantages of Stanza’s tagging. YS contributed to the calculation of precisions, recalls, and F-scores of the lexico-grammatical features. YL contributed to the gold labels of the lexico-grammatical features and the analysis of the (dis)advantages of Stanza’s tagging. All authors contributed to the article and approved the submitted version.
